# Effect of Low-Frequency Modulation on the Penetration Profile and Microstructure in Double-Pulsed GMAW of AA6061 Aluminum Alloy

**DOI:** 10.3390/ma19214455

**Published:** 2026-10-23

**Authors:** Zhanhui Zhang, Lai Zhou, Dong Chen, Wenbo Liao, Shenggui Chen, Yu Li, Pan Qi

**Affiliations:** 1 School of Future Technology, Guangzhou Polytechnic University, No. 1342 Shiliang Road, Panyu District, Guangzhou 511483, China; zhangzh@gzpyp.edu.cn (Z.Z.);; 2 School of Art and Design, Guangzhou Polytechnic University, Guangzhou 511483, China; 3 Institute of Advanced Materials and Technology, Guangzhou Polytechnic University, Guangzhou 511483, China

**Keywords:** double-pulsed GMAW, AA6061 aluminum alloy, weld bead profile, molten pool, penetration fluctuation, grain morphology

## Abstract

**Highlights:**

- Weld penetration fluctuates periodically once the number of pulses per group reaches 7 or more; the onset of the fluctuation is bracketed between 8.33 Hz and 5.95 Hz.
- Peak/valley penetration difference reaches 532.06% of the reinforcement difference.
- Droplet impingement and pool-bottom constraint are proposed to sharpen the penetration valleys and to refine the grains along the upslope edges.

**Abstract:**

Double-pulsed gas metal arc welding (DP-GMAW), in which strong and weak pulse groups alternate periodically at a low frequency, is promising for improving the weld quality of aluminum alloys. However, how the low-frequency modulation shapes the bead profile and microstructure remains unclear. Bead-on-plate DP-GMAW of AA6061 aluminum alloy was therefore performed, in which the numbers of pulses in the strong and weak groups were varied from 1:1 to 30:30 (low frequency 41.67–1.39 Hz). Fish-scale ripples appeared on the bead surface at all tested frequencies, whereas the penetration fluctuated periodically only when the number of pulses per group reached 7 or more, so that the onset of the periodic fluctuation is bracketed between 8.33 Hz and 5.95 Hz. Both the penetration and the reinforcement peak/valley differences increased as the low frequency decreased; the largest penetration peak/valley difference reached 664.33 μm at 2.78 Hz, and the ratio of the penetration to the reinforcement peak/valley difference was largest at 3.79 Hz, where the penetration difference (627.83 μm) amounted to 532.06% of the reinforcement difference, suggesting that the droplet-impingement-driven flow affects the pool bottom far more strongly than the bead surface. At 3.79 Hz, the valley and peak angles reached 91.52° and 158.33°, respectively. It is proposed that the droplet-driven melt erodes the solid front wall at weak-to-strong transitions and forms sharp valleys, while the unconstrained flow preserves blunt peaks. Columnar grains formed along the downslope edges, whereas the grains were refined along the upslope edges. These results provide a quantitative basis for tailoring the weld profile and microstructure via low-frequency current modulation.

## 1. Introduction

Owing to their low density, high specific strength, good corrosion resistance and excellent formability, aluminum alloys have become indispensable structural materials in aerospace, rail transit, automotive and shipbuilding industries, where weight reduction is a primary concern [1,2,3]. Among them, the Al–Mg–Si alloy AA6061 is one of the most widely used medium-strength 6xxx-series alloys, and its joining quality directly determines the service reliability of the corresponding structures. Gas metal arc welding (GMAW), featuring high deposition rate, high efficiency, strong adaptability and low cost, is the mainstream fusion welding process for manufacturing large aluminum alloy structures [4]. However, during fusion welding of aluminum alloys, defects such as porosity, coarse columnar grains and solute segregation are easily introduced into the weld metal, and the softened heat-affected zone (HAZ) caused by dissolution and coarsening of the strengthening precipitates considerably degrades the joint strength [3,5,6]. Considerable efforts have therefore been devoted to porosity suppression and quality improvement, for example, through power-modulated laser welding, magnetic-field-assisted laser-arc hybrid welding and other energy-field-assisted processes [7,8,9].

Pulsed GMAW controls the heat input and the droplet transfer by modulating the peak and base currents. On this basis, double-pulsed GMAW (DP-GMAW) superimposes a low-frequency modulation (typically 0.5–10 Hz) on the pulsed current, so that strong and weak pulse groups alternate periodically [4,10]. The periodic stirring of the molten pool promotes pore escape, refines the solidification structure, reduces porosity and produces regular fish-scale ripples on the bead surface [10,11,12,13]. DP-GMAW and its variants (e.g., double-wire DP-GMAW and ultrasonic-frequency double-pulsed arc) have been successfully applied to the welding and wire-arc additive manufacturing of various aluminum alloys, such as 6061, 6082, 2319 and 2219 alloys, yielding finer grains, lower porosity and higher joint strength [14,15,16,17,18,19]. In double-wire and variable-polarity variants of DP-GMAW, the influence of the weak-pulse peak current on droplet transfer [20] and the associated arc characteristics [21] have also been clarified in recent years, and the beneficial effect of high-frequency or ultrasonic-frequency pulse modulation on droplet transfer, penetration and pore elimination has been further demonstrated [5,22,23].

The weld bead profile is governed by the heat transfer and fluid flow in the molten pool, in which droplet impingement, arc pressure, electromagnetic force, buoyancy and Marangoni convection are the dominant driving forces [24,25]. Numerical studies have shown that, in the radial velocity component of the pool flow, the effects of droplet impingement and the Marangoni force are large, while in the vertical velocity component, the droplet impingement effect is dominant, and the alloying elements transported by droplet impingement accumulate at the bottom of the fusion zone [24]. The Marangoni flow further modifies the temperature gradient, cooling rate and solidification microstructure [26,27,28], and the flow pattern of the pool determines the pool length and the local cooling conditions [27]. These studies indicate that the liquid metal flow, especially the flow driven by droplet impingement, plays a decisive role in the formation of the penetration profile.

Although DP-GMAW has been extensively studied, most published studies focus on arc characteristics, porosity reduction and mechanical properties [4,10,22], and the microstructural refinement produced by the low-frequency modulation has been reported mainly in terms of average grain size measured over the whole weld zone [10,18]. In particular, Liu et al. [14] have already shown for AA6061 that double-pulse modulation produces a periodically varying longitudinal penetration together with characteristic spatial microstructural features, and Zhang et al. [29] have clarified how the constraint imposed by the weld pool wall governs the bead profile in GMAW of aluminum alloys. However, these studies did not quantify how the number of pulses within the strong/weak pulse groups, i.e., the low-frequency period, controls the amplitudes and the peak/valley angles of the longitudinal penetration fluctuation, nor did they compare the grain morphology along the upslope and downslope edges of a single penetration peak or valley. The specific novelty of the present work relative to [14,29] is therefore threefold: (i) the systematic quantification of how the number of pulses per group, i.e., the low-frequency period, controls the amplitudes and the peak/valley angles of the longitudinal penetration fluctuation; (ii) the quantitative correspondence between the current waveform and the longitudinal profile, including the sharp-valley/blunt-peak asymmetry at the weak-to-strong and strong-to-weak transitions; and (iii) the within-specimen comparison of the grain morphology along the upslope and downslope edges of a single penetration valley, which provides localized microstructural evidence for the flow asymmetry inferred from the profiles. In GMAW of aluminum alloys, the weld profile has been shown to be governed by the flow pattern of the liquid metal, which is determined not only by the driving forces but also by the constraint imposed by the weld pool wall [29]; on this basis, profile maps of weld beads covering the overflowing, matching and inadequate stages have been established, and the formation of the reinforcement profile has been related to the change in the flow pattern [30]. The impingement of droplets has likewise been found to control both the weld profile and the grain morphology, since the grains along the wall of the finger-like penetration are refined by the violent flow driven by droplet impingement, whereas curved columnar grains emerge on the side not subjected to the impact [31]. In particular, the longitudinal penetration profile of DP-GMAW welds exhibits characteristic sharp valleys and blunt peaks corresponding to the weak-to-strong and strong-to-weak current transitions, but the formation mechanism, especially the respective contributions of droplet impingement and of the constraint imposed by the molten-pool bottom wall, has not been clarified. In arc-welded aluminum alloys, the grain structure is governed by heterogeneous nucleation, dendrite fragmentation and the columnar-to-equiaxed transition [32]. Moreover, because the same flow that shapes the penetration profile also controls the local cooling and solidification conditions at the pool bottom, the grain morphology is expected to differ between the upslope and downslope edges of a penetration peak or valley, yet this has rarely been reported.

The objective of this study is to quantitatively clarify how the low-frequency modulation period controls the number of pulses within the strong and weak pulse groups and controls the longitudinal penetration profile and the grain morphology at the pool bottom of DP-GMAW welds, and to infer from the profile and microstructure observations the respective roles of droplet impingement and pool-bottom constraint in forming the sharp valleys and blunt peaks. To this end, bead-on-plate DP-GMAW of 3 mm thick AA6061 plates with ER4047 filler wire was carried out, in which the numbers of pulses in the strong and weak pulse groups were varied from 1:1 to 30:30, corresponding to low frequencies of 41.67–1.39 Hz. The current/voltage waveforms, the bead appearance, the longitudinal cross-section profiles and the characteristic dimensions (penetration peak/valley, penetration peak/valley angles, reinforcement peak/valley and peak-to-peak distance) were systematically analyzed. The formation mechanism of the periodic penetration fluctuation is then inferred from the combined effects of droplet impingement and the constraint imposed by the pool bottom wall, and the grain morphology along the upslope and downslope edges of the penetration peaks and valleys was further characterized by metallographic observation. This study provides a quantitative basis for tailoring the longitudinal profile and the microstructure of aluminum alloy welds through low-frequency current modulation, and the quantitative profile descriptors established here can also serve as process targets for data-driven welding optimization [33,34,35].

## 2. Materials and Methods

### 2.1. Base Metal, Filler Metal and Welding Parameters

Bead-on-plate welding was performed on AA6061 aluminum alloy plates (250 mm × 60 mm × 3 mm) using ER4047 filler wire; the experimental setup is shown in Figure 1a, and the chemical compositions of the base metal and the filler wire are listed in Table 1, which provide a basis for evaluating weld dilution [36]. The chemical compositions given in Table 1 were determined by energy-dispersive spectroscopy (EDS), as described in our previous study [36]. The base metal was used in the as-received temper condition (T6), and the plate surface to be welded was degreased and mechanically ground to remove the surface oil contamination and the oxide film before welding. The filler wire diameter was 1.2 mm, the wire stick-out was 12 mm, the torch angle was 90°, and the contact-tip-to-work distance was 11 mm; welding was carried out using a self-developed digital double-pulsed MIG inverter power source. A stable pulsed GMAW process was employed, in which the average current can be varied over a wide range by adjusting the base current; the single-pulse parameters of the strong and weak pulse groups are listed in Table 2. Pure argon was supplied coaxially as the shielding gas at a flow rate of 18 L/min, and the welding speed was 10 mm/s. The heat input *P* in Table 2 was calculated using Equation (1), where *η* is the thermal efficiency, typically taken as 0.7–0.8 for MIG welding. Considering the favorable heat dissipation conditions, a value of 0.7 was adopted here. *U* and I denote the welding voltage and current, respectively, and *v* is the welding speed. The welding voltage was maintained at 24.3 V in all experiments.

*P* = *ηUI*/*v* (1)

### 2.2. Double-Pulse Experiments

To clarify the influence of double-pulsed welding on the formation of the weld bead profile, DP-GMAW experiments with different numbers of pulses within the pulse groups were carried out in this work. The schematic diagram of the current waveform is shown in Figure 1b. To accurately compare the influence of the strong and weak pulse groups on the weld bead profile and the microstructure, and to exclude the interference of other factors as much as possible, the number of pulses in the strong pulse group was kept identical to that in the weak pulse group, i.e., N1 = N2. The specific current waveform parameters used in this work are listed in Table 3; the single-pulse parameters of the strong and weak pulse groups are the same as those in Table 2. Here, the low-frequency period T listed in Table 3 is defined as the total duration of one strong pulse group plus one weak pulse group, i.e., T = 2N × (peak time + base time), where N is the number of pulses within each group; with the single-pulse parameters of Table 2 (2 ms + 10 ms = 12 ms per pulse), this gives T = 24 × N ms and a low frequency of f = 1/T, so that N = 1 corresponds to T = 24 ms and f = 41.67 Hz. The low-frequency periods and frequencies listed in Table 3 are calculated on this basis. For each parameter set, three independent bead-on-plate welds were produced under identical conditions, and three longitudinal sections were prepared for the profile and metallographic analyses; the contour dimensions reported in Section 3 were measured several times under the stereomicroscope and averaged, and the reading-to-reading scatter was small compared with the specimen-to-specimen differences discussed below, so that it did not affect the comparative conclusions.

### 2.3. Metallographic Preparation

The metallographic analyses served two purposes: (i) to reveal the longitudinal penetration profile and to measure the bead contour dimensions (penetration peak/valley, penetration peak/valley angles, reinforcement peak/valley and peak-to-peak distance) defined in Section 3.1.1, and (ii) to characterize the grain morphology along the upslope and downslope edges of the penetration peaks and valleys at the pool bottom. After welding, transverse cross-section specimens of the welds were cut by wire electrical discharge machining (WEDM). Since the aluminum alloy specimens were relatively soft, they were successively ground with SiC abrasive papers of #500, #1000, #2000, #5000, and #7000 grits, and then polished with diamond spray polishing agents of W5.0, W2.5, W1.5, and W0.5 particle sizes in sequence. After polishing, the specimens were etched with Keller’s reagent (2.5 mL HNO_3_ + 1.5 mL HCl + 1 mL HF + 95 mL H_2_O) for 20–30 s to reveal the metallographic microstructure of the cross-sections. The macroscopic morphology of the welds was first examined with an LIOO SZ850T (LIOO Optics, Beijing, China) stereo microscope, and then the microstructure of the welds was characterized with an Olympus GX51 optical microscope (Olympus Corporation, Tokyo, Japan).

## 3. Results and Discussion

### 3.1. Effect of Droplet-Impingement Modulation by Double Pulses on the Weld Bead Profile

#### 3.1.1. Characteristics of the Weld Bead Profile

To investigate the weld bead profile characteristics under the combined action of droplet impingement and wall constraint, bead-on-plate experiments were carried out in this section by changing the number of pulses within the strong and weak pulse groups, and the specific experimental parameters were as described in Section 2.2. Figure 2 shows the current and voltage waveforms acquired for different numbers of pulses in the pulse groups, in which the number of pulses within each pulse group gradually increased from 1 to 30. It can be seen from these figures that both the current and the voltage exhibit good repeatability, and neither of them drops to zero, indicating that the welding process proceeds stably in all cases.

Figure 3 shows the weld bead appearance and the longitudinal cross-section morphology of the welds produced by double pulses with different numbers of pulses in the pulse groups. It can be seen that when the number of pulses within a pulse group is 5 or fewer, i.e., the low frequency is 8.33 Hz or higher, regular fish-scale ripples already appear on the weld bead surface, but no regular fluctuation at the same frequency appears in the weld penetration, as shown in Figure 3a–e. When the number of pulses within a pulse group reaches 7 or more, i.e., the low frequency decreases to 5.95 Hz and below, the weld penetration begins to exhibit regular periodic fluctuations, as shown in Figure 3f–k.

To better characterize the evolution of the weld bead morphology, the dimensions of the inner contour within the longitudinal section were statistically measured. Taking specimen 13# as an example, the meanings of the measured parameters, such as the penetration peak/valley, the penetration peak/valley angle, the reinforcement peak/valley and the peak-to-peak distance, are illustrated in Figure 4. In Figure 4 and Figure 5, the penetration peak and the penetration valley denote the deepest and the shallowest points of the fusion line within one modulation cycle, respectively, and the corresponding peak angle and valley angle are measured at these two points; the legend entry “Bottom” in Figure 5b,c refers to this penetration valley.

Figure 5 shows the contour dimensions of the longitudinal sections of the double-pulsed welds at different frequencies, and Figure 5a shows the correspondence between the specimen identifiers and the low frequencies. As can be seen from Figure 3, the onset of the periodic penetration fluctuation is bracketed between 8.33 Hz (5:5, no fluctuation) and 5.95 Hz (7:7, periodic fluctuation), i.e., the fluctuation appears when the number of pulses within a pulse group reaches 7 or more, as indicated by the dashed boxes. In this work, a weld is regarded as exhibiting periodic penetration fluctuation when at least three consecutive penetration peaks and valleys repeat along the longitudinal section at a spacing consistent with the low-frequency period of Table 3, i.e., when the measured peak-to-peak distance corresponds to the distance travelled by the welding torch during one modulation cycle (v/f). By this criterion, specimens 20# and 30# also belong to the periodically fluctuating regime; however, the quantitative analysis in the following is restricted to specimens 7#, 11#, 13# and 15#, in which the periodic fluctuation is most regular. All peaks and valleys are completely captured within the observed longitudinal section. In comparison, specimens 20# and 30# are shown in Figure 3j,k for completeness but are not included in the statistical comparison of Figure 5, because for specimens 20# and 30#, the higher energy of the strong pulse groups caused complete penetration of the base metal: the true penetration capability exceeded the plate thickness, so that the 3 mm plates used here could no longer reflect the actual penetration depth of these two welds. The weld bead contour dimensions defined in Figure 4 of these four specimens are statistically measured, with the results shown in Figure 5b–f. As can be seen from Figure 5b, both the penetration peak and the penetration valley increase with the number of pulses within the pulse groups, but the rate of increase gradually slows down, and the difference between the penetration peak and valley shows the same trend, with a maximum value of 664.33 μm obtained for specimen 15#. As for the peak/valley angles, the penetration valley angle first increases and then decreases, while the penetration peak angle exhibits only a slight fluctuation; however, the difference between the two angles is huge, and the maximum difference between the penetration peak and valley angles is 66.81°, obtained for specimen 11#, as shown in Figure 5c. For the weld reinforcement, its surface height also varies with the pulse frequency, and its maximum difference also occurs for specimen 15#, but the difference is only 217.92 μm, as shown in Figure 5d, which is far smaller than the penetration peak/valley difference. To clearly compare the penetration peak/valley difference with the reinforcement peak/valley difference, Figure 5e gives the comparison of the two, from which it can be seen that both differences reach their maxima for specimen 15#, but their ratio is the largest for specimen 11#, i.e., when the low frequency is 3.79 Hz, the penetration peak/valley difference is as high as 532.06% of the reinforcement peak/valley height difference, namely 627.83 μm. Meanwhile, the peak-to-peak distance of the penetration also increases with the number of pulses within the pulse groups, as shown in Figure 5f.

#### 3.1.2. Formation Mechanism of the Weld Bead Profile

It should be noted that the penetration profiles, the current/voltage waveforms and the metallographic appearances presented in this study are direct experimental observations, whereas the droplet-driven flow and the pool-bottom interaction discussed below are interpretations inferred from these observations. The above differences in the longitudinal weld profiles are mainly related to the heat input, the droplet impingement and the wall constraint regulated by the current waveform. In GMAW, both the wall constraint of the weld pool and the impingement of droplets play decisive roles in determining the flow pattern of the liquid metal and hence the weld profile [29,31]. Regarding the penetration peak/valley difference, as the number of pulses within the pulse groups increases, the durations of both the strong and weak pulse groups become longer. Since the strong pulse group inputs more heat, it corresponds to a deeper penetration, and likewise the weak pulse group corresponds to a shallower penetration; both of them become deeper or shallower with the increase in the number of pulses in the strong and weak pulse groups. As for the peak-to-peak distance of the weld, with the increase in the number of pulses within the pulse groups, i.e., with the prolongation of the durations of both the strong and weak pulse groups, the peak-to-peak distance increases when the welding speed remains unchanged.

It is worth noting that although the numbers of pulses in the strong and weak pulse groups are identical, it can be clearly seen from Figure 5c that the penetration peak/valley angles show a significant difference between “sharp” and “blunt”. When the low frequency is 3.79 Hz, the corresponding penetration peak angle is as large as 158.33°, which is about 173.00% of the corresponding sharper penetration valley angle of 91.52°. For the same transition between pulse groups of different energy, the reason why the peak angle is much larger than the valley angle is attributed to the different combined actions of droplet impingement and wall constraint. Figure 6 shows the correspondence between the strong/weak pulse groups together with their transition points and the longitudinal weld profile. As can be seen from the figure, during the strong pulse group stage, which corresponds to the higher current level enclosed by the black box in the figure, the weld penetration keeps increasing; during the weak pulse group stage, which corresponds to the lower current level enclosed by the green box, the weld penetration keeps decreasing; when the current changes from the strong pulse group to the weak pulse group, the penetration peak angle with a larger angle corresponds to this transition, as indicated by the yellow box; while when the current changes from the weak pulse group to the strong pulse group, a penetration valley angle with a smaller angle corresponds to it, as indicated by the purple box.

The waveform is registered to the longitudinal section by converting the welding time into the welding distance through the constant welding speed (10 mm/s), taking the same time origin for the waveform acquisition and the welding process; owing to the thermal inertia of the molten pool, the profile response lags slightly behind the current transition, and the boxes in Figure 6 mark the corresponding stages schematically. The scale bar denotes 1000 μm. Physically, during the strong pulse group stage, the energy input is higher, so the capability of melting the base metal is stronger and the penetration gradually increases; once the current enters the weak pulse group, the lower energy input weakens the melting capability and the penetration gradually decreases. Consequently, the penetration reaches its maximum at the transition from the strong pulse group to the weak pulse group, where it stops increasing and then turns to decrease; conversely, it reaches its minimum at the transition from the weak pulse group to the strong pulse group, where it stops decreasing and then turns to increase.

When the welding current changes from the weak pulse group to the strong pulse group, as described above, the penetration of the molten pool corresponding to the weak pulse group is shallow. Therefore, after the current enters the strong pulse group, the penetration gradually increases due to the increase in current; based on the profile and waveform observations, the evolution of the pool is interpreted as follows, and the process of the increase in pool depth is shown in Figure 7a, in which the red-shaded region is a local schematic of the pool bottom as the welding torch travels forward. With the continuous accumulation of the heat input of the strong pulse group, more and more base metal at the bottom wall is melted, so that a contour boundary with gradually increasing penetration is formed, as shown by the black dashed line in Figure 7a. It can be seen that after the droplet impinges on the pool bottom, the bottom in its traveling direction is unmelted solid base metal; constrained by the solid base metal, the droplet flows backward and downward along the pool bottom wall. Because of the increased depth, the downward and backward directions are also surrounded by solid base metal, so that the molten metal carrying a large amount of heat and momentum moves downward, melting the solid base metal and increasing the weld penetration, while the molten metal flowing backward melts the rear solid base metal. In this way, the liquid metal driven by the droplet impingement continuously “sharpens” the solid base metal located between it and the previous pool, and such continuous motion finally forms a penetration valley with a small angle.

When the welding current changes from the strong pulse group to the weak pulse group, as described above, the penetration of the pool corresponding to the strong pulse group is large. After the current enters the weak pulse group, the penetration decreases due to the decrease in current, and the trajectory of the pool evolution is shown in Figure 7b. Unlike the transition from the weak to the strong pulse group, since the pool formed in the preceding strong pulse group stage is deeper, after the droplet impinges on the pool, although it still flows backward and downward under the constraint of the solid base metal in its traveling direction, its lower and rear parts are the not-yet-solidified pool formed in the previous stage of the strong pulse group. Therefore, during its backward and downward movement, it is no longer constrained by solid base metal, so that it can continue to flow along the previous smooth pool boundary. Consequently, when the pool boundary is not subjected to the violent melting action of the fluid, the smooth transitional boundary formed is preserved, and thus the large angle of the penetration peak is preserved.

Meanwhile, it is worth noting that Figure 5e also compares the penetration peak/valley difference and the reinforcement peak/valley difference in the different specimens, and the specific values are listed in Table 4. It can be seen from the table that for the same specimen, the difference between the penetration peak and valley is significantly larger than that between the reinforcement peak and valley; even for the minimum value within the studied range, the penetration difference is more than three times the reinforcement difference. This suggests that the influence of the droplet-driven liquid flow on the weld penetration is significantly greater than that on the reinforcement. The ratio therefore serves as a comparative indicator of the different sensitivity of the constrained pool bottom and the free pool surface to the periodic modulation; because both regions are also shaped by the overall weld-pool configuration, it is not used here as a direct proof of the flow mechanism. It must be emphasized, however, that the final bead geometry and the 532.06% amplitude ratio alone cannot quantify how the droplet heat and momentum are distributed between the pool bottom and the pool surface; the following interpretation is therefore put forward as a hypothesis rather than as a conclusion. The preferential response of the pool bottom is consistent with the fact that the droplets transfer their mass, superheat and kinetic energy directly onto the pool bottom, whereas the pool surface is a free surface that is continuously replenished by the overflowing liquid metal and is therefore much less sensitive to the periodic change in the group energy [24,29,31]. Nevertheless, competing thermal explanations cannot be excluded on the basis of the present measurements alone: (i) the strong and weak pulse groups differ in average current (104 A versus 72 A) and in heat input (177 J/mm versus 122 J/mm), so that part of the penetration difference may simply reflect the larger integrated heat input of the strong group rather than a stronger local momentum transfer; (ii) the periodic variation in the arc pressure and of the arc-root position with the current level may modulate the pool surface and the pool depth independently of the droplet momentum [24,25]; and (iii) the surface-tension-driven Marangoni convection, whose direction and intensity depend on the local temperature gradient and on the surface-active solute content, may redistribute heat along the pool surface and thereby smooth the reinforcement fluctuation [26,27,28]. Separating these contributions quantitatively would require a validated transient model of the pool flow or independent in situ observations of the molten pool [24,27]. A more conservative reading of the same data, namely that the penetration is intrinsically more sensitive than the reinforcement to any periodic perturbation of the energy input, is equally consistent with Table 4 and cannot be ruled out.

### 3.2. Grain Morphology Along the Upslope and Downslope Edges

The alternation between the strong and weak pulse groups described above not only gives rise to the difference in the penetration peak/valley angles but is also reflected in the microstructure of the weld. Figure 8 shows the grain morphology at the bottom of the molten pool for the different specimens, in which Figure 8a shows the microstructure of specimen 15# on both sides of a penetration valley angle; here, the contour along which the penetration becomes shallower in the welding direction is defined as the “upslope edge”, and the opposite contour is defined as the “downslope edge”. Figure 8b and Figure 8c are, respectively, the magnified views of the grain morphology along the downslope and the upslope edges of Figure 8a. A comparison of the two figures reveals that the grain morphology differs markedly between the two edges: obvious columnar grains are present along the downslope edge, whereas along the upslope edge the columnar grains disappear and the grains are markedly refined. It should be noted that the features discussed here are identified as grains on the basis of the grain-boundary contrast revealed by Keller’s reagent etching under optical microscopy; under this etching condition, the outlined units correspond to the grain structure of the weld metal rather than to individual dendritic or cellular substructures, which is the common practice for the optical characterization of aluminum alloy weld metals [31,32,37]. Nevertheless, it should be emphasized that the evidence presented here is internally controlled and therefore does not hinge on absolute grain-size statistics: the claim made is a categorical one, namely whether or not the columnar growth along a given edge is interrupted by the periodically reversed local flow, rather than a numerical mean grain size, and it is established by a within-specimen comparison that is repeated across specimens, and that disappears in step with the periodic fluctuation of the penetration, as discussed below. Indeed, the enhanced grain refinement in DP-GMAW has been elaborated in previous studies in terms of the remelting of the previously solidified region during the strong pulse groups and the strong periodic stirring of the molten pool: Wang et al. [38] demonstrated by a coupled heat-transfer and fluid-flow analysis that double-pulsed modulation periodically varies the local cooling rate and the solidification growth rate at a constant average heat input, thereby refining the weld microstructure, while Chen et al. [39] showed experimentally that the pool oscillation and the shear force associated with each thermal pulse can fragment the growing dendrites and provide abundant nuclei for equiaxed growth. Moreover, the present comparison itself contains a built-in internal control: the upslope and downslope edges compared in Figure 8a–c belong to the same penetration valley of the same specimen and therefore share the same nominal composition, thermal cycle and etching condition, so that the local flow history is the only systematic difference between them; in addition, the same columnar-versus-refined contrast recurs in specimens 13# and 7# and disappears systematically once the number of pulses per group falls to 5 or fewer (Figure 8h–j), i.e., exactly when the periodic fluctuation of the penetration vanishes. The physical reason for this difference is that, as described above, when the current switches to the weak pulse group, i.e., during the process in which the contour becomes shallower along the upslope edge, the droplet impinges on the bottom of the molten pool and, constrained by the solid base metal in front of the pool, continuously strikes and stirs the previously solidified zone while moving backward and downward, so that the growth of the grains is repeatedly interrupted and they cannot grow into columnar grains, as shown in Figure 8c. In this respect, the results obtained here are consistent with previous findings that the grains along the wall of the finger-like penetration are markedly refined by the violent flow driven by droplet impingement [31]. Alternative explanations for this morphological asymmetry, such as a local difference in the heat-flow direction or in the growth orientation of the columnar grains between the two edges, cannot be entirely excluded on the basis of optical metallography alone; however, such explanations would apply symmetrically to the two edges of the same valley, whereas the observed asymmetry recurs in specimens 13# and 7# and disappears in step with the vanishing of the periodic penetration fluctuation, which favors the flow-related interpretation. Compared with the established knowledge that droplet impingement can influence the grain morphology [31,38,39], the new information provided here is the strict spatial correlation between the grain morphology and the two sides of a single penetration valley: the same flow feature that sharpens or preserves the profile boundary also leaves a localized microstructural signature on the corresponding edge. When the welding current switches to the strong pulse group, i.e., during the process along the downslope edge, the upward flow of the liquid metal in the pool is weakened under the combined action of the constraint of the solid base metal wall at the rear side and of gravity, so that the grains located above this region on the rear side can grow continuously into columnar grains without being stirred by droplet impingement, as shown in Figure 8b. The same applies to the other specimens, such as 13# and 7#. It should be noted, however, that this difference gradually decreases as the number of pulses within the pulse groups decreases, and that once the number of pulses decreases to 5 or fewer, the difference in grain morphology also disappears gradually, because no obvious peak/valley difference remains in the penetration at the bottom of the pool, as shown in Figure 8h–j.

## 4. Conclusions

(1) For AA6061 aluminum alloy bead-on-plate welding by double-pulsed GMAW with equal numbers of pulses in the strong and weak pulse groups (N1:N2 = 1:1–30:30, low frequency 41.67–1.39 Hz), the welding process remained stable, with neither the current nor the voltage dropping to zero. Regular fish-scale ripples appeared on the weld surface at all low frequencies; however, the weld penetration began to fluctuate periodically with the strong/weak pulse groups only from 7 pulses per group onwards, so that the onset of the periodic fluctuation is bracketed between 8.33 Hz (5:5, no fluctuation) and 5.95 Hz (7:7, periodic fluctuation). Both the penetration peak/valley difference and the reinforcement peak/valley difference increased as the low frequency decreased; the penetration peak-to-peak distance also increased with the number of pulses, and the maximum penetration peak/valley difference of 664.33 μm was obtained for specimen 15#.

(2) For the double-pulse-modulated current waveform, the ratio of the penetration peak/valley height difference to the reinforcement peak/valley height difference reaches its maximum when the low frequency is 3.79 Hz, at which the penetration peak/valley height difference is about 532.06% of the reinforcement peak/valley height difference. That is to say, when the pool bottom fluctuates violently, only slight differences appear in the ripples on the upper surface of the pool, suggesting that the flow of the liquid metal affects the pool bottom and the weld reinforcement to different degrees, and that this effect is the largest at the pool bottom and then decays with increasing height.

(3) In double-pulsed welding, the penetration peak/valley angles also vary correspondingly with the transition between the strong and weak pulse groups. When the current changes from the weak pulse group to the strong pulse group, the resulting penetration valley angle is small; conversely, when the current changes from the strong pulse group to the weak pulse group, the resulting penetration peak angle is large. The ratio of the penetration peak angle to the valley angle reaches its maximum of 173.00% at the low frequency of 3.79 Hz.

(4) The periodic alternation of the strong and weak pulse groups is also reflected in the solidification microstructure at the bottom of the pool. Along the downslope edges, the grains grow continuously into obvious columnar grains because the upward flow of the liquid metal is weakened and the grains are no longer stirred; along the upslope edges, in contrast, the columnar grains disappear, and the grains are markedly refined, and this asymmetry is attributed to the flow driven by droplet impingement, which is inferred to strike and stir the previously solidified zone and thereby to interrupt grain growth repeatedly; within the present study, these microstructural differences serve as qualitative supporting evidence for the inferred flow asymmetry. As the number of pulses within the pulse groups decreases, this microstructural difference gradually diminishes, and once the number decreases to 5 or fewer, it disappears together with the periodic fluctuation of the penetration.

## Figures and Tables

**Figure 1:**
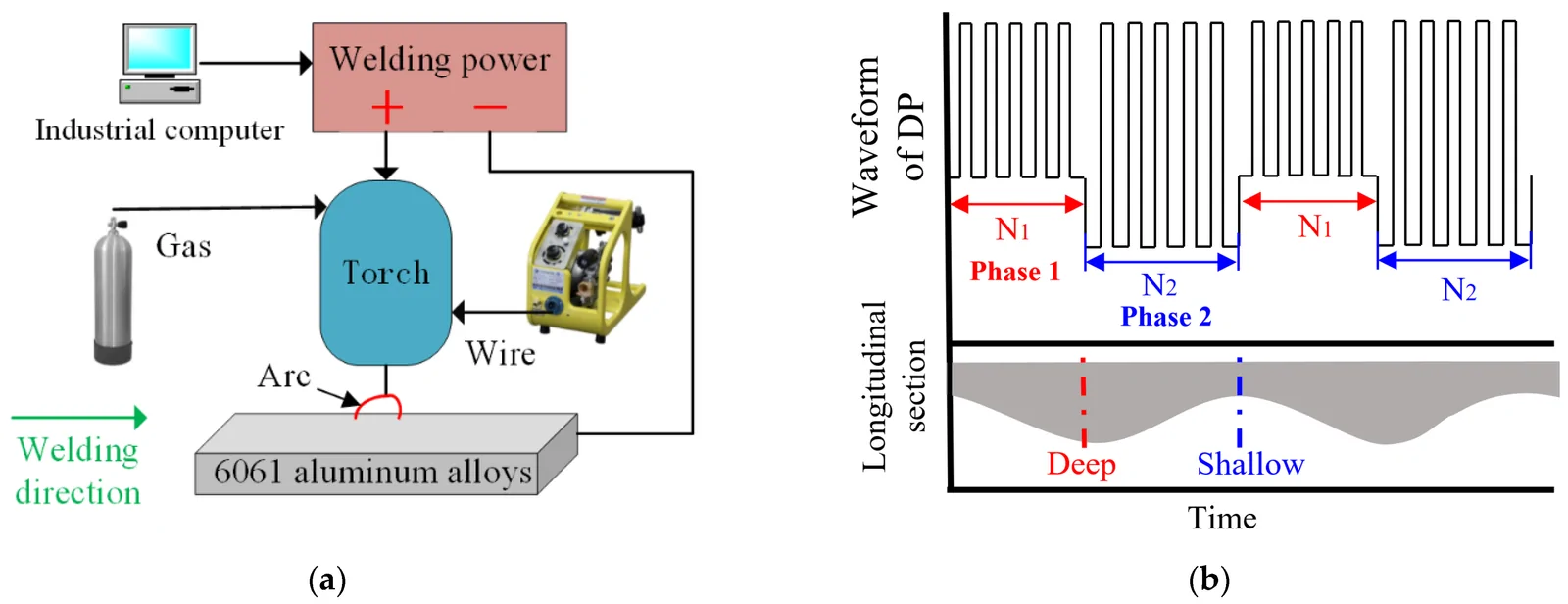
Experimental setup and the current waveform for DP-GMAW: (**a**) experimental setup; (**b**) the adjustment of the current waveform for DP-GMAW.

**Figure 2:**
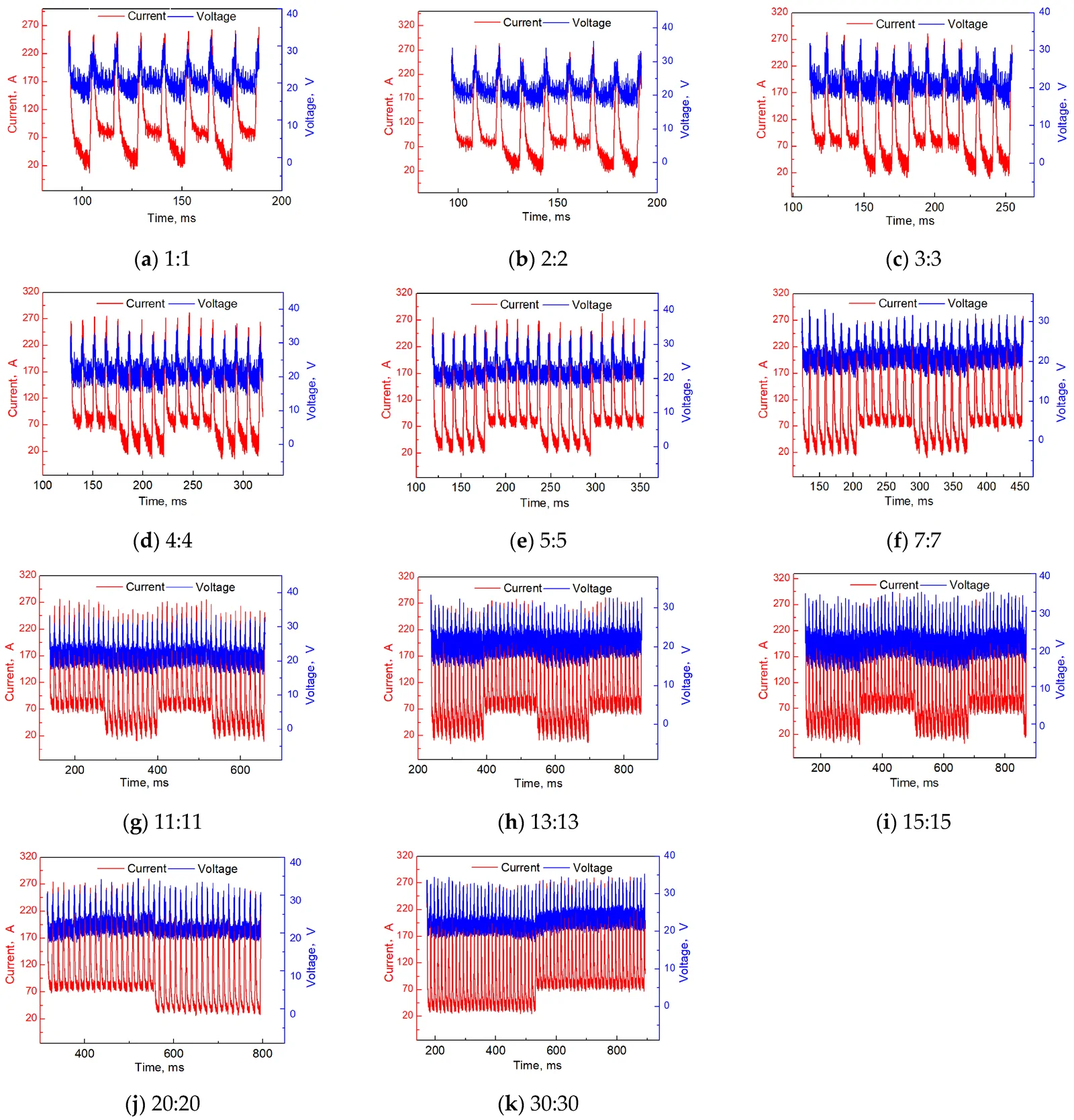
Measured welding current (red) and arc voltage (blue) waveforms of the DP-GMAW welds, plotted against welding time, for the different numbers of pulses in the strong and weak pulse groups (specimens of Table 3): (**a**) 1:1 (1#); (**b**) 2:2 (2#); (**c**) 3:3 (3#); (**d**) 4:4 (4#); (**e**) 5:5 (5#); (**f**) 7:7 (7#); (**g**) 11:11 (11#); (**h**) 13:13 (13#); (**i**) 15:15 (15#); (**j**) 20:20 (20#); (**k**) 30:30 (30#). The alternation between the strong and weak pulse groups is reflected in the periodic envelopes of both curves, and neither the current nor the voltage drops to zero, indicating that the welding process proceeds stably in all cases.

**Figure 3:**
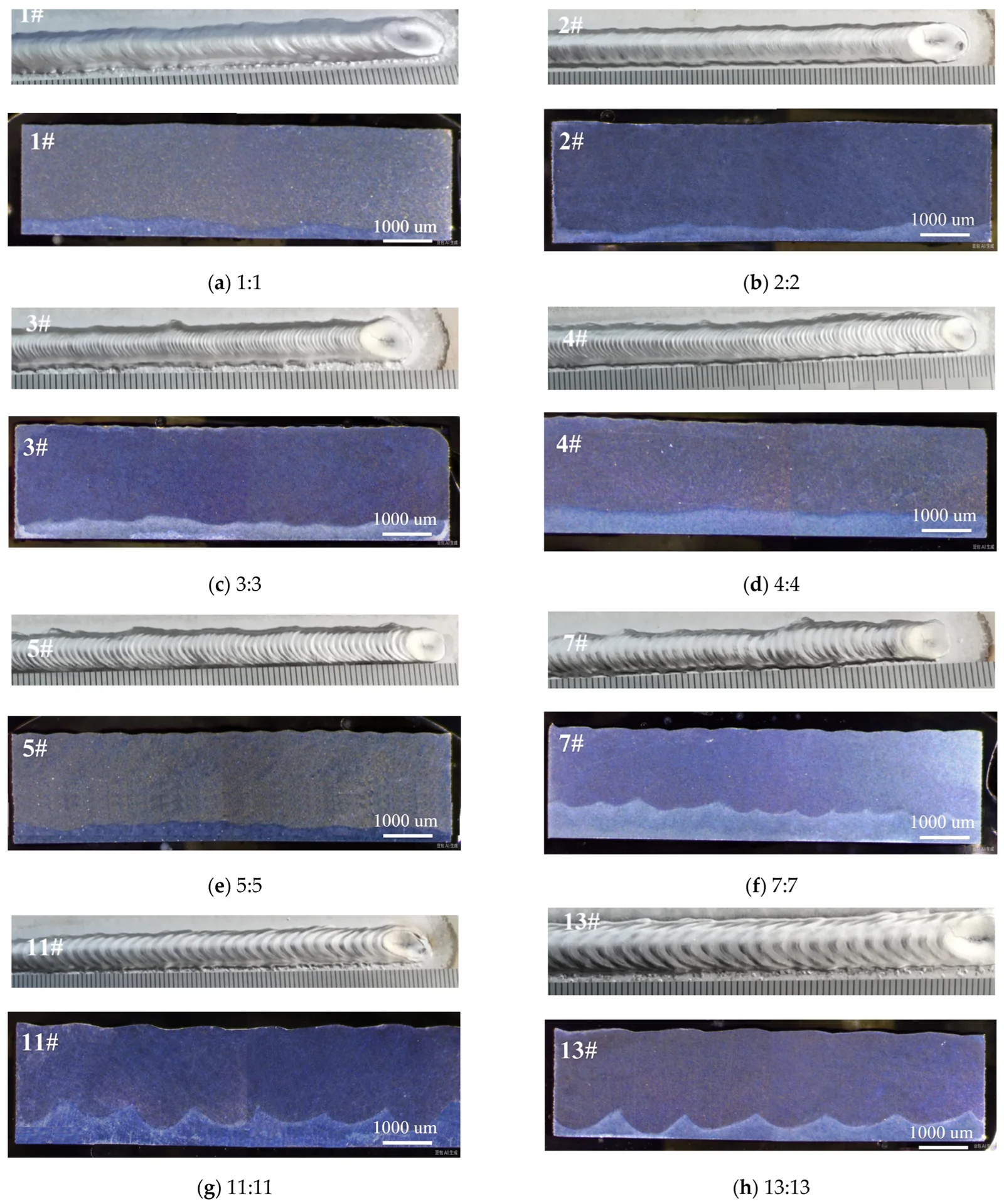

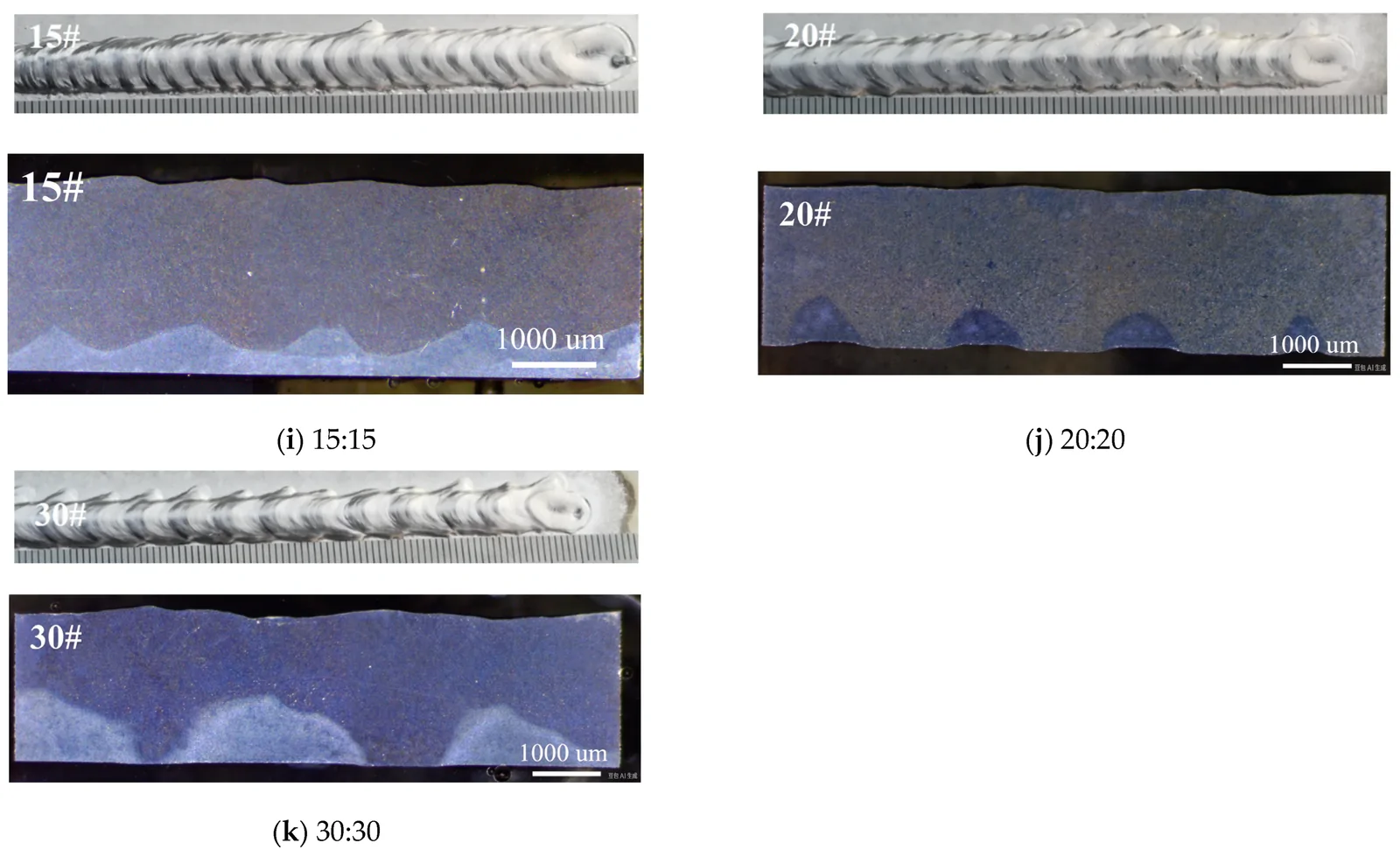
Weld bead appearance (upper image of each panel) and the corresponding longitudinal cross-section profile (lower image of each panel) of the double-pulsed welds at different low frequencies: (**a**) 1:1 (1#, 41.67 Hz); (**b**) 2:2 (2#, 20.83 Hz); (**c**) 3:3 (3#, 13.89 Hz); (**d**) 4:4 (4#, 10.42 Hz); (**e**) 5:5 (5#, 8.33 Hz); (**f**) 7:7 (7#, 5.95 Hz); (**g**) 11:11 (11#, 3.79 Hz); (**h**) 13:13 (13#, 3.21 Hz); (**i**) 15:15 (15#, 2.78 Hz); (**j**) 20:20 (20#, 2.08 Hz); (**k**) 30:30 (30#, 1.39 Hz). Regular fish-scale ripples are present on the bead surface in all cases, whereas a periodic fluctuation of the penetration appears only from panel (**f**) onwards. The scale bars in the bead-appearance images and the longitudinal macrographs denote 1000 μm (1 mm).

**Figure 4:**
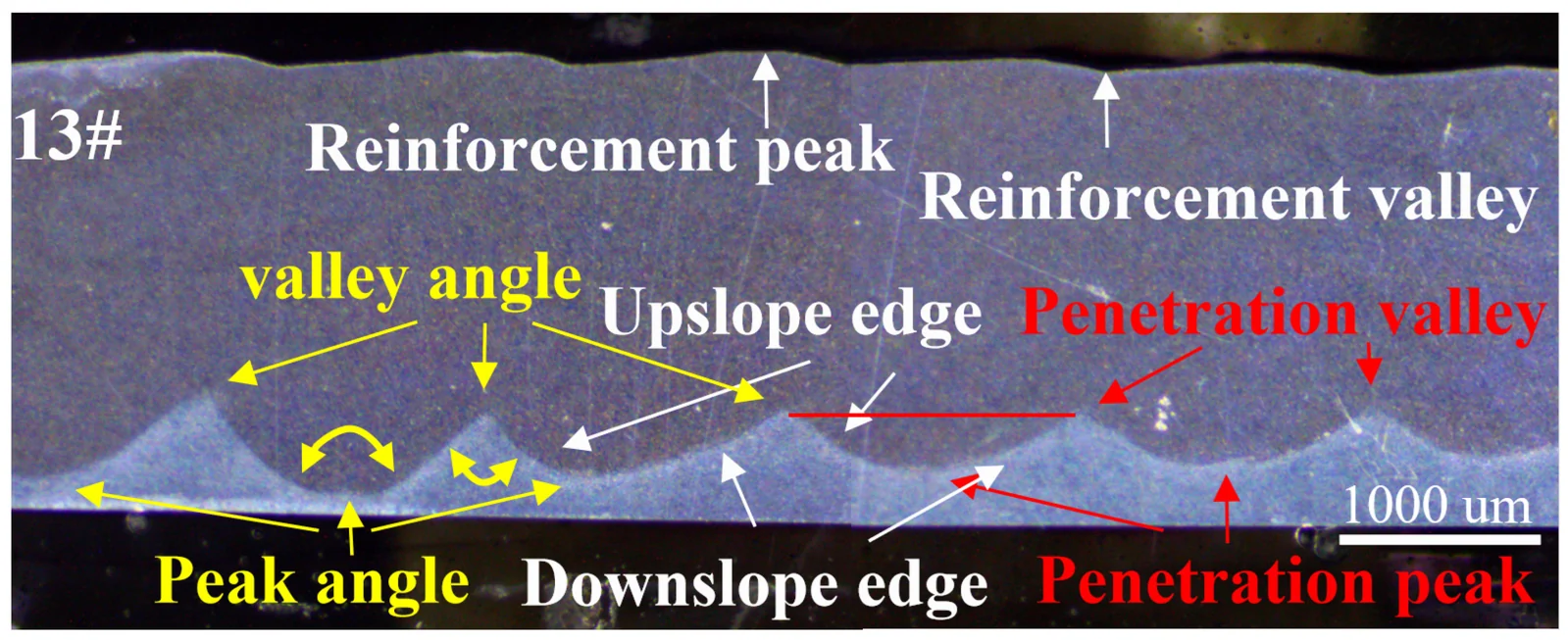
Definition of the measurement parameters of the longitudinal section, illustrated on the longitudinal macrograph of specimen 13# (13:13, 3.21 Hz): penetration peak, penetration valley, penetration peak angle and valley angle, upslope edge and downslope edge of the fusion line, and reinforcement peak and reinforcement valley of the bead surface. The scale bar denotes 1000 μm.

**Figure 5:**
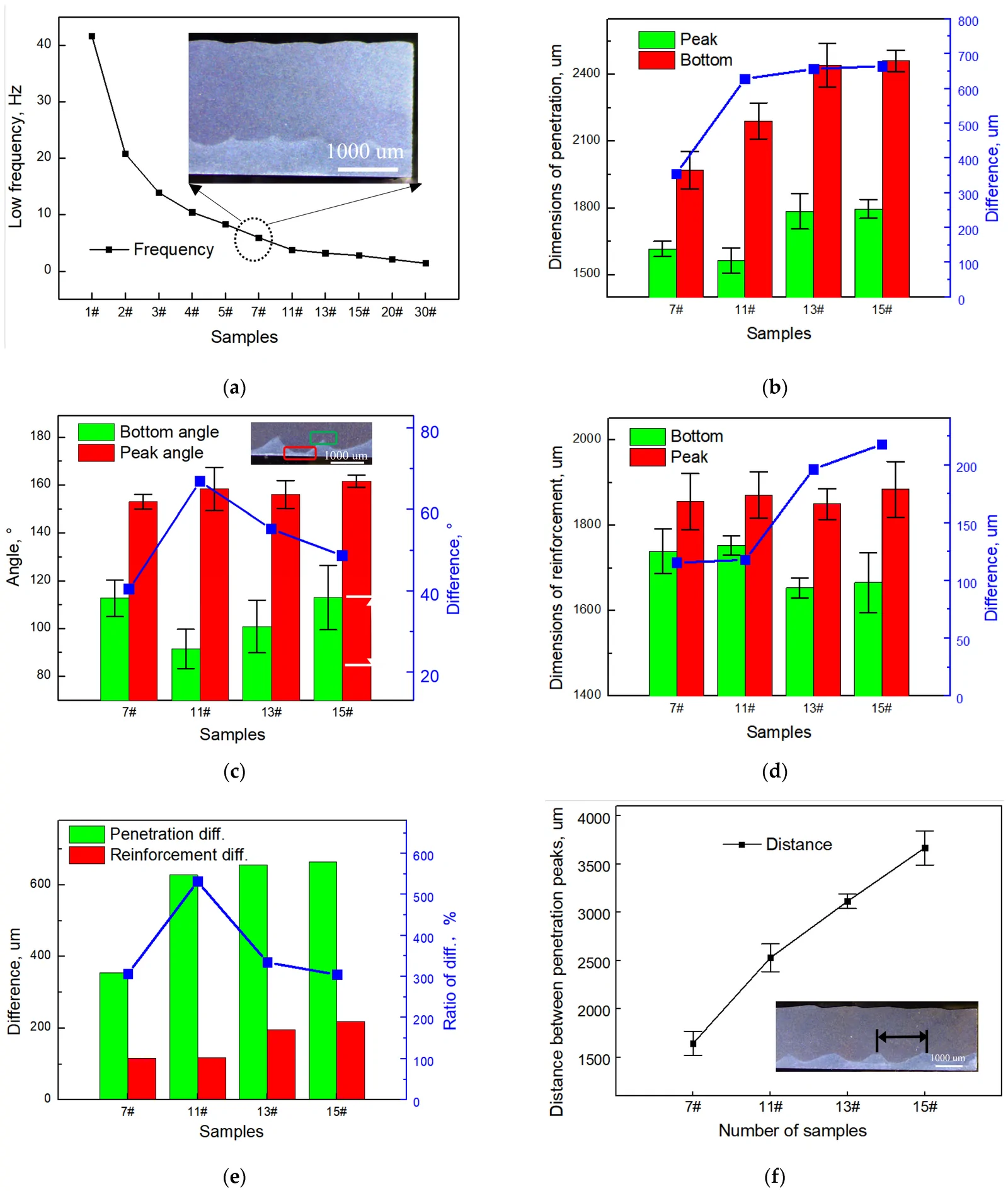
Contour dimensions of the longitudinal sections of the double-pulsed welds, measured for the periodically fluctuating specimens 7#, 11#, 13# and 15# (abscissa: specimen identifier as listed in Table 3; the axis labels “Samples” in (**a**) and “Number of samples” in (**b**–**f**) denote this specimen identifier): (**a**) correspondence between the specimen identifier and the low frequency; (**b**) penetration peak (“Peak”) and penetration valley (“Bottom”), together with their difference (right axis); (**c**) penetration valley angle (“Bottom angle”) and penetration peak angle (“Peak angle”), together with their difference (right axis); (**d**) reinforcement peak and reinforcement valley; (**e**) penetration peak/valley difference compared with the reinforcement peak/valley difference; (**f**) peak-to-peak distance of the penetration. In (**b**,**c**), the legend entry “Bottom” denotes the penetration valley, consistent with the terminology of Figure 4. The insets in (**a**,**c**,**f**) show longitudinal macrographs with scale bars of 1000 μm. Error bars denote the scatter of the measured values among the three measurement groups taken along the longitudinal sections of each specimen, with three welds prepared for each parameter set.

**Figure 6:**
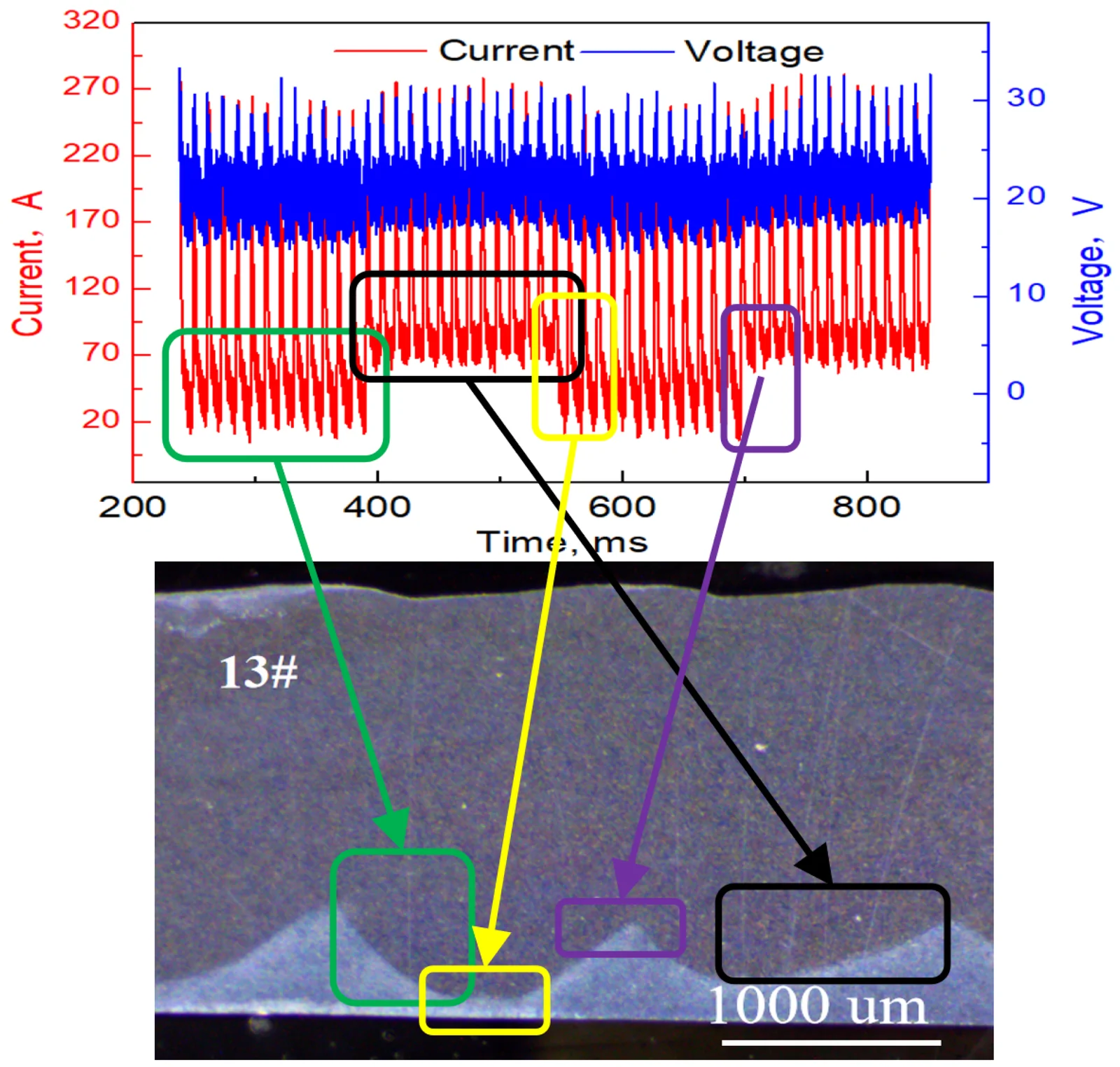
Correspondence between the measured current waveform ((**upper panel**); red: welding current, blue: arc voltage) and the longitudinal weld bead profile (**lower panel**) of the double-pulsed weld. The black box marks the strong pulse group (higher average current), during which the penetration increases; the green box marks the weak pulse group (lower average current), during which the penetration decreases; the yellow box marks the penetration peak formed at the strong-to-weak current transition; and the purple box marks the penetration valley formed at the weak-to-strong current transition.

**Figure 7:**
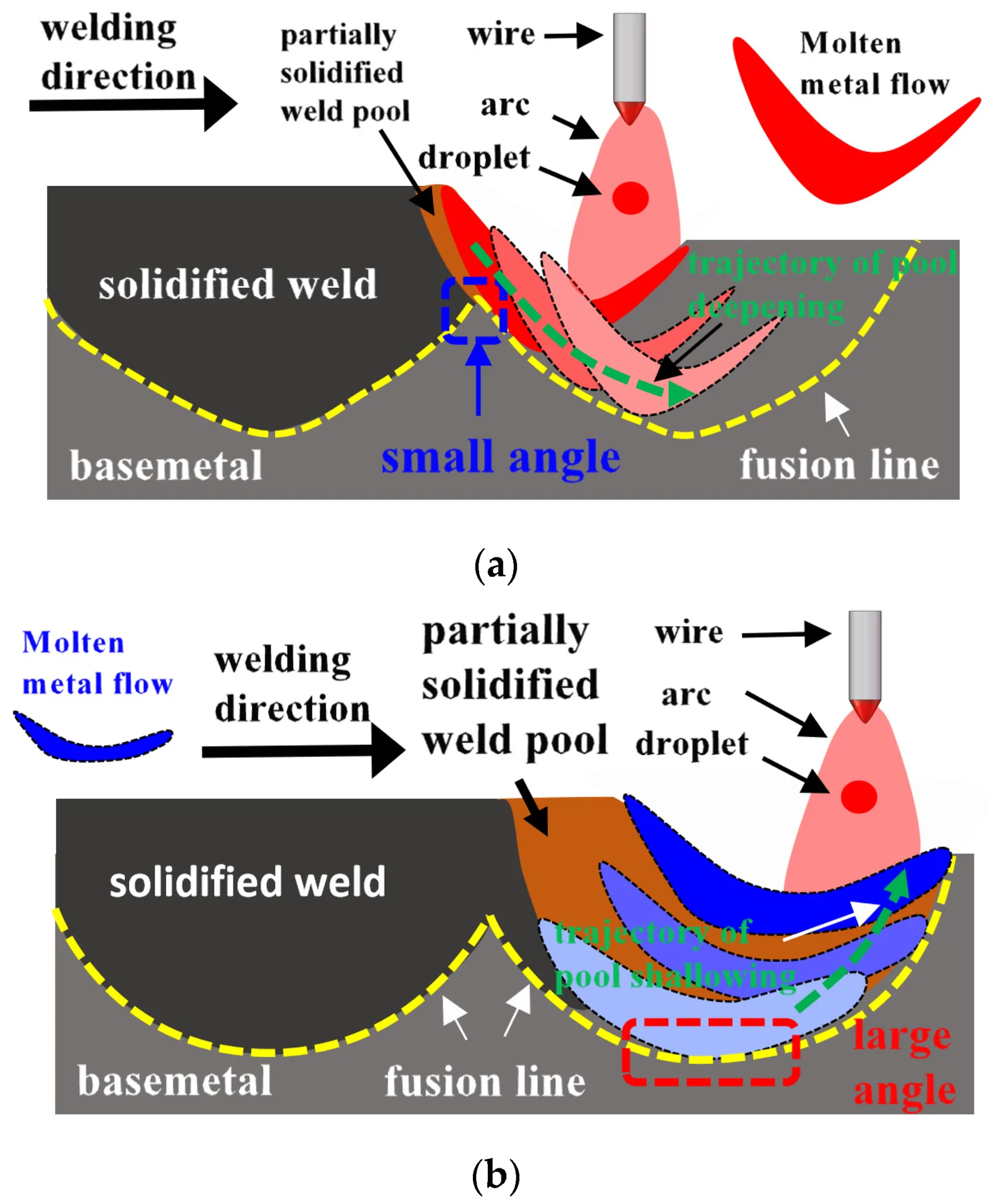
Schematic of the weld profile formation in DP-GMAW: (**a**) deepening process of the molten pool; (**b**) shallowing process of the molten pool.

**Figure 8:**
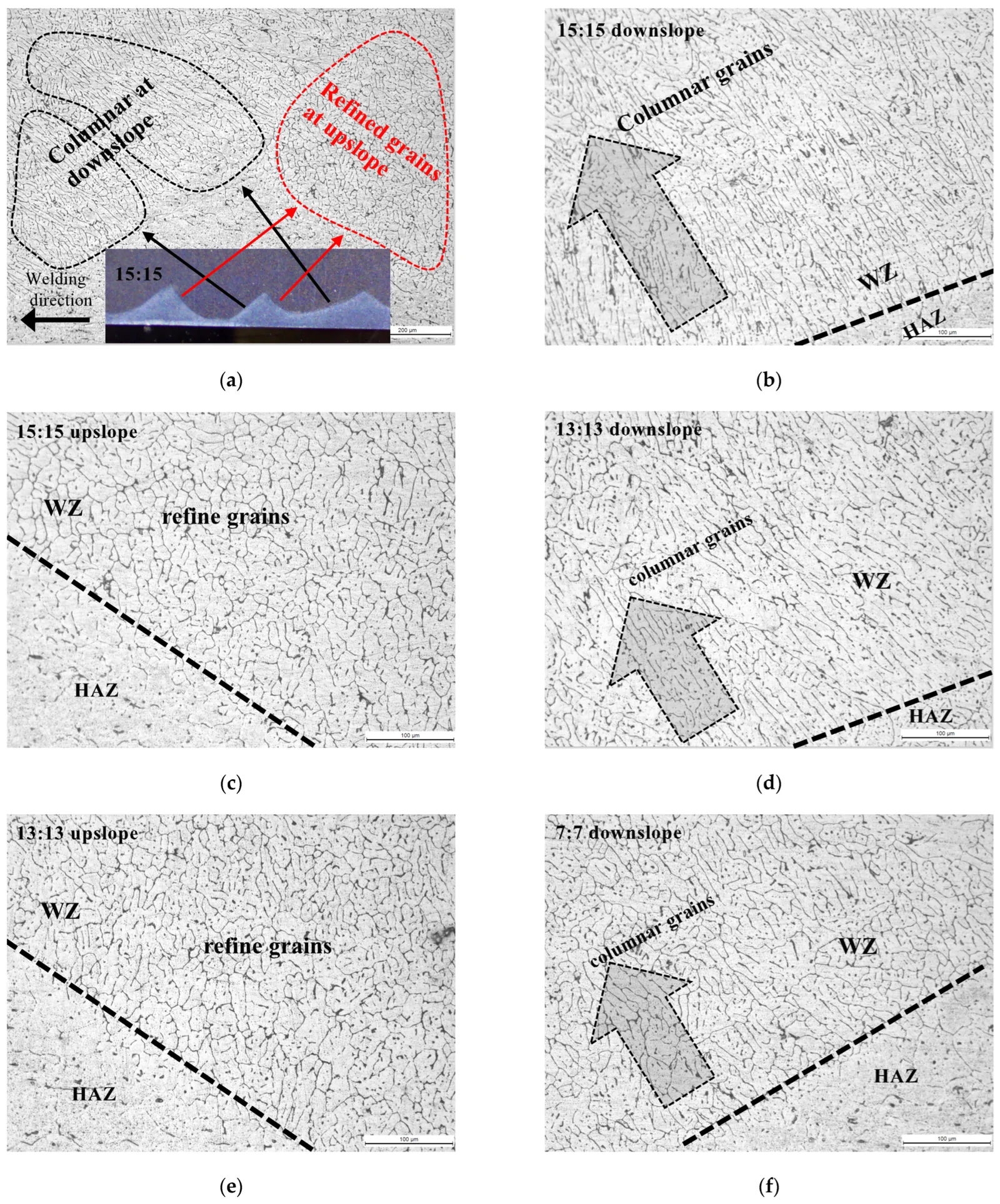

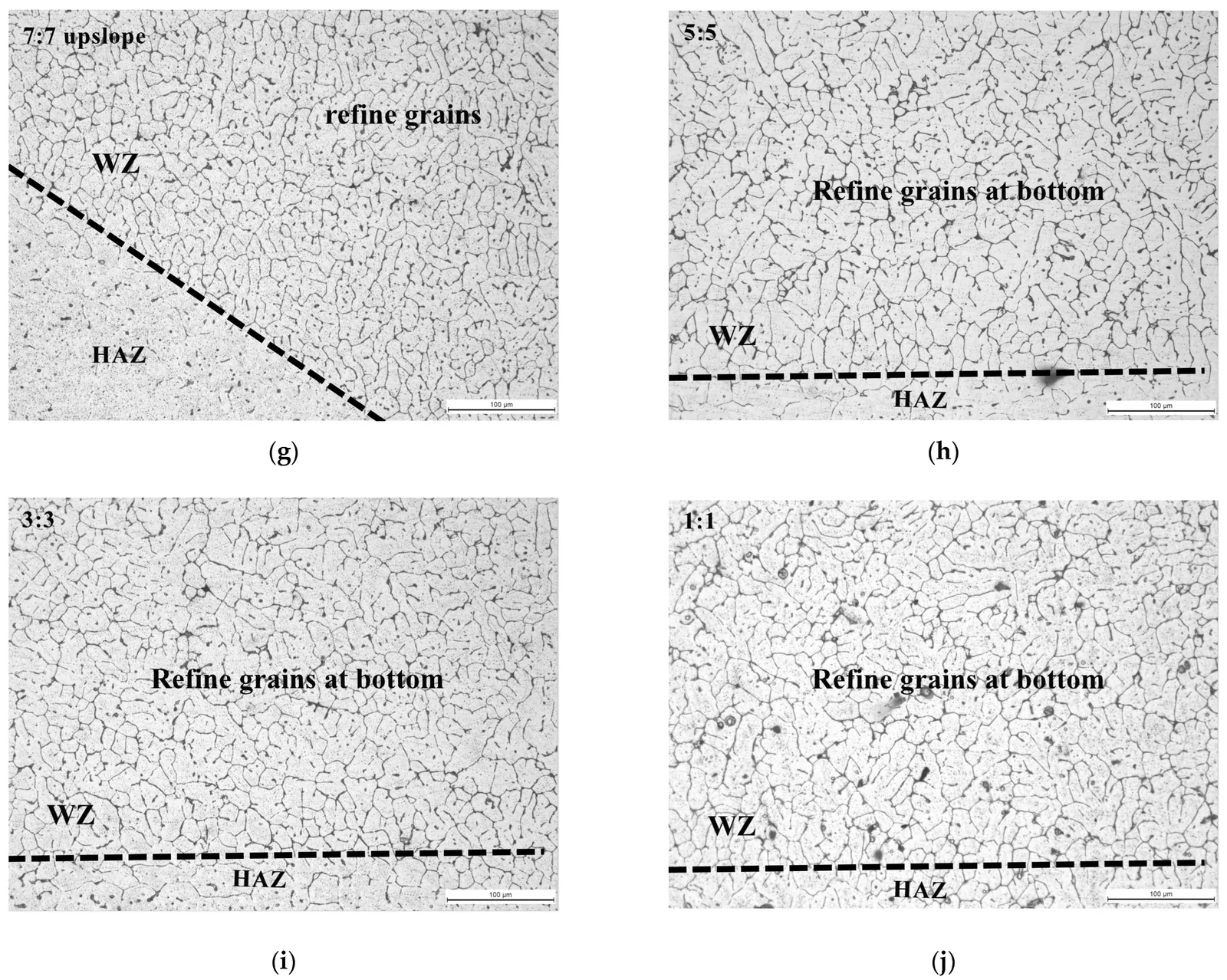
Grain morphology at the bottom of the molten pool of the different specimens, revealed by Keller’s reagent etching under an optical microscope (the welding direction is indicated by the arrow in (**a**), and a scale bar is given in each panel): (**a**) overall view of specimen 15# (15:15) showing the regions on both sides of a penetration valley; (**b**) magnified view of the downslope edge of 15#; (**c**) magnified view of the upslope edge of 15#; (**d**) downslope edge of 13#; (**e**) upslope edge of 13#; (**f**) downslope edge of 7#; (**g**) upslope edge of 7#; (**h**) 5#; (**i**) 3#; (**j**) 1#. The regions outlined by the dashed lines in (**a**–**c**) mark the columnar grains along the downslope edge and the refined grains along the upslope edge.

**Table 1:** Chemical compositions of AA6061 and ER4047 (wt.%) [29].

Material	Mg	Si	Fe	Cu	Mn	Cr	Ti	Al
AA6061	0.87	0.66	0.42	0.29	0.09	0.27	0.08	Bal.
ER4047	0.05	11.06	0.30	0.13	0.12	0.04	0.07	Bal.

**Table 2:** Single-pulse parameters of the strong and weak pulse groups.

Specimen	Peak Current (A)	Peak Time (ms)	Base Current (A)	Base Time (ms)	Average Current (A)	Heat Input (J/mm)
B30	280	2	30	10	72	122
B70	280	2	70	10	104	177

**Table 3:** DP-GMAW experiments with different numbers of pulses in the pulse groups.

Specimen	Number of Pulses in the Strong Pulse Group	Number of Pulses in the Weak Pulse Group	Low-Frequency Period (ms)	Low Frequency (Hz)
1#	1	1	24	41.67
2#	2	2	48	20.83
3#	3	3	72	13.89
4#	4	4	96	10.42
5#	5	5	120	8.33
7#	7	7	168	5.95
11#	11	11	264	3.79
13#	13	13	312	3.21
15#	15	15	360	2.78
20#	20	20	480	2.08
30#	30	30	720	1.39

**Table 4:** Penetration peak/valley differences and reinforcement peak/valley differences in the double-pulsed welds.

Specimen	Penetration Difference (μm)	Reinforcement Difference (μm)	Penetration Difference/Reinforcement Difference
7#	354.27	115.67	3.06
11#	627.83	118	5.32
13#	656.07	196.25	3.34
15#	664.33	217.92	3.05

## Data Availability

The original contributions presented in this study are included in the article. Further inquiries can be directed to the corresponding author.

## Abbreviations

The following abbreviations are used in this manuscript:

DP-GMAW	Double-pulsed gas metal arc welding
GMAW	Gas metal arc welding
HAZ	Heat-affected zone
WEDM	Wire electrical discharge machining
